# Ethylene: A Modulator of the Phytohormone-Mediated Insect Herbivory Network in Plants

**DOI:** 10.3390/insects15060404

**Published:** 2024-06-01

**Authors:** Leonel Tarcisio da Cristina Bungala, Chanung Park, José Eulário Lampi Dique, Ramaraj Sathasivam, Su Young Shin, Sang Un Park

**Affiliations:** 1 Department of Crop Science, Chungnam National University, 99 Daehak-ro, Yuseong-gu, Daejeon 34134, Republic of Korea; tarcisiodacristina@gmail.com (L.T.d.C.B.); chanungpark92@gmail.com (C.P.); ramarajbiotech@gmail.com (R.S.); 2 Mozambique Agricultural Research Institute, Central Regional Center, Highway N° 6, Chimoio P.O. Box 42, Mozambique; diquejosedique4@gmail.com; 3 Department of Biology, Natural Science Institute, Federal University of Lavras, Lavras 37203-202, Brazil; 4 Using Technology Development Department, Bio-Resources Research Division, Nakdonggang National Institute of Biological Resources (NNIBR), 137, Donam 2-gil, Sangju-si 37242, Republic of Korea; 5 Department of Smart Agriculture Systems, Chungnam National University, 99 Daehak-ro, Yuseong-gu, Daejeon 34134, Republic of Korea

**Keywords:** ethylene, phytohormones, insect herbivory, plant defence

## Abstract

**Simple Summary:**

According to recent research, insect herbivory can be positively and negatively regulated by ET biosynthesis and perception. This review explores the defence mechanisms of plants against insect herbivory, including biosynthesis and signal transduction of ET. Furthermore, it discusses the role of ET in triggering defences against herbivory, the ET interaction with other phytohormones, and the external application of ET stimulation.

**Abstract:**

Plants have evolved to establish insect herbivory defences by modulating their metabolism, growth, and development. Precise networks of phytohormones are essential to induce those herbivory defences. Gaseous phytohormone ET plays an important role in forming herbivory defences. Its role in insect herbivory is not fully understood, but previous studies have shown that it can both positively and negatively regulate herbivory. This review presents recent findings on crosstalk between ET and other phytohormones in herbivory responses. Additionally, the use of exogenous ETH treatment to induce ET in response to herbivory is discussed.

## 1. Introduction

Plants challenge herbivores in two ways: directly, by affecting host plant preferences or survival and reproductive achievement (direct defence), and indirectly, through other species that are natural enemies of insect pests (indirect defence) [1,2]. Various environmental stressors activate multiple signal transduction pathways to safeguard plants with an efficient spatial and temporal defence response [3]. For this reason, plants must be capable of recognising and prioritising each signalling pathway to assemble the most efficient defence and minimise possible damage [4]. Plants use constitutive and induced defences to protect themselves from pathogen and pest assault and to resist and adapt to such stresses [5]. Phytohormones play a significant role in regulating specific physiological and biochemical processes to induce the formation of a complex defence network [6]. Ethylene (ET) is a gaseous hormone that regulates plant development and stresses. Abiotic stresses, such as osmotic and heat, and biotic stresses from fungi and insects induce the production of ET in plants [7]. In particular, ET plays an essential role in plant defences by synthesising defensive volatiles and phenolics, allowing plants to resist pathogens by regulating genes responsible for immunity [8,9]. ET has been studied in many aspects, but studies addressing its role as an inductor of defence resistance against herbivory stresses in crops have yet to draw attention. However, the responses to insect herbivory, whether ET’s independent role or its intricate crosstalk with other phytohormones, raise crucial questions. This review is a call to action, aiming to provide a comprehensive overview of scientific data addressing the pivotal role of ET in plant responses to insect herbivory. The first part of this review delves into the diverse strategies that plants can employ to defend themselves against insect herbivory. We then shift our focus to the intricate process and mechanism of ET biosynthesis and signal transduction in response to herbivory in plants.

Moreover, we delve into the concept of ET crosstalk with other phytohormones, particularly JA, and we present the latest findings on using ETH as an ET inducer. When these signalling pathways are integrated, plants can efficiently allocate resources and maintain a balance between response and defence. By exploring the role of ET and its crosstalk with other phytohormones, we aim to not only stimulate further research and innovation in entomological science but also make a significant contribution to the promotion of sustainable agriculture practices.

Our methodological approach, which includes a quantitative and qualitative synthesis, is more than just a theoretical exercise. It is a practical tool that allows us to review published evidence related to ET and its potential role in plant defence mechanisms and responses to insect herbivory. In order to evaluate a topic using contemporary bibliometrics or meta-analyses, a traditional bibliometric study is essential [10]. This is beneficial for identifying research trends, interests, and variables such as author affiliations. Additionally, a comparative analysis of scientific productivity can also be provided by this tool at the national, institutional, or individual level [11].

We conducted an extensive literature search using keywords such as “ET and insect herbivory”, “ET and biotic stress”, “ET and pest management”, and “ET biosynthesis and signalling” across multiple databases from 11 July to 23 August 2023. The criteria for inclusion were stringent, involving articles published in the last 20 years (from 2003 to 2023). Only articles published in international journals and indexed in Web of Science or Scopus were considered. Moreover, the selected manuscripts for online inspection had to be in English. After rigorous screening, considering this study’s aim, only 110 entries were identified and included in the bibliometric analysis, representing a focused and manageable dataset for analysis.

## 2. Plant Defence against Insect Herbivory

**Figure 1:**
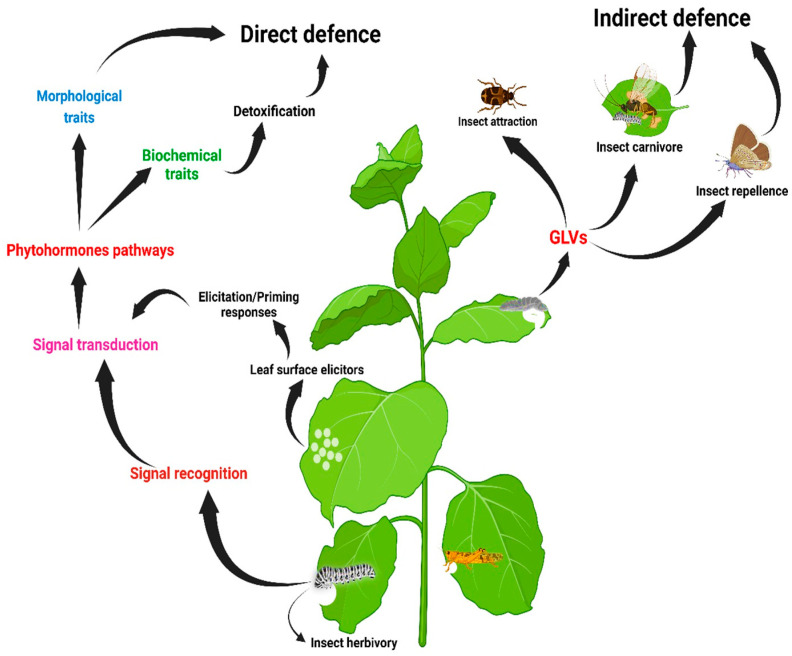
A brief overview of plant defences against insect herbivory (adapted from [18]). Abbreviation: GLVs, green leaf volatiles.

Over time, plants have developed several mechanisms to protect from, defend against, and tolerate insect herbivory [12,13]. Upon perceiving insect herbivory, plants adjust their responses through a flexible and finely adjusted network that stimulates precise defence responses [14]. For efficient responses, plants must be capable of recognising these attacks with high accuracy via intracellular signalling. They must convert these signals into suitable physiological, cellular, and biochemical responses [15]. Once the herbivory is perceived, the identified signals are transduced through a network of multifaceted signalling transduction pathways, possibly leading to gene expression changes and, finally, the production of defence chemicals (Figure 1) [16,17]. For example, plants may release volatile organic compounds as a response to herbivory and, depending on the type of insect feeding [18,19], different defence signalling pathways are activated, producing specific volatile compounds [18]. These volatile compounds produced by herbivory negatively affect herbivores and may attract insect predators and parasitoids, which may help reduce the herbivore population [20]. Non-volatile compounds like flavonoids have been cited as internal plant messengers, and their importance as weapons against insect herbivory has been described [21].

For example, studies have shown that plant *POLYPHENOL OXIDASE (PPO)* genes, which synthesise toxic quinones, are closely related to stress, insect damage, disease, and microbial invasion [22,23]. One study found alterations in the expression of defence-related genes such as *PHLOEM PROTEIN 2 (PP2)* and *WRKY* transcription factors in sorghum in response to the sugarcane aphid (*Melanaphis sacchari*) [24]. A study on herbivory transcription analysis found the induction of *PHENYLALANINE AMMONIA-LYASE (PAL)* in both aphid-susceptible and -resistant sorghum [25]. Orthologues of *Arabidopsis ETHYLENE INSENSITIVE 3 (EIN3)* and *Arabidopsis ETHYLENE RESPONSE SENSOR 2 (ERS2)* in sorghum were induced during infestation [25]. This indicates that the herbivory-induced defence may be strongly connected to ET and other phytohormones.

## 3. ET Biosynthesis, Signal Transduction, and the Response to Herbivory

**Figure 2:**
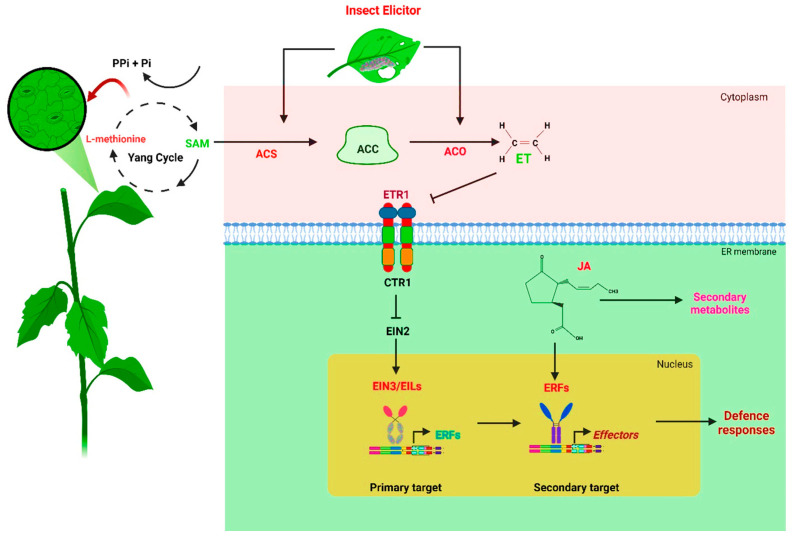
Review of ET-mediated insect herbivory signalling and transduction pathways. Insect elicitors and stimuli upregulate *ACS* and *ACO,* increasing ET biosynthesis. CTR1 is deactivated after ET binds to its receptor ETR1. Therefore, EIN2 is accumulated and signals positively downstream to the nuclear transcription factors EIN3/EIL. Transcription factor *ERFs* are expressed by EIN3/EIL, and ERFs activate further ethylene response genes. A defence response by ERFs is also activated with the help of JA. As a result, defence metabolites and proteins are synthesised upon activating the response gene. An arrow indicates a positive interaction, while a bar indicates a negative one. Abbreviations: ACS, 1-aminocyclopropane-1-carboxylic (ACC) synthase; ACO, ACC oxidase; ET, ethylene; JA, jasmonic acid; ETR1, Ethylene Receptor 1; CTR1, Constitutive Triple Response 1; EIN2, Ethylene Insensitive 2; EIN3, Ethylene Insensitive 3; EILs, Ethylene Insensitive 3-likes; ERFs, Ethylene Response Factors.

Plants respond to insects and wounding in complex ways, involving multiple signalling pathways and extensive transcriptional reprogramming [26,27]. ET is a gaseous phytohormone essential for seed germination, rooting, blooming, fruiting, and senescence, as well as the plant’s response to numerous biotic and abiotic stimuli and its ability to resist such stresses [28]. Once the stress is perceived, it induces ET production, and the perception of ET leads to signal transduction, resulting in observable responses [29,30]. The multistep mechanisms behind ET biosynthesis and signalling represent many levels of control of its production and subsequent response [31]. It is well-known that plant growth and development are regulated by ET signalling, and the intensity and period of stress can affect the quantity of ET produced [32,33]. S-adenosyl-L-methionine (SAM) is the first step in plants’ biochemical route to synthesise ET [34,35]. SAM is converted to ACC (1-aminocyclopropane-1 carboxylic acid) by the enzyme ACC SYNTHASE (ACS), which is known as the most crucial step in ET biosynthesis, and oxidation of ACC by ACC OXIDASE (ACO) produces ET [36]. In the presence of stress, ET biosynthesis genes such as *ACS* and *ACO* are transcriptionally and post-translationally upregulated, thus stimulating ET biosynthesis (Figure 2) [37,38]. *ACS* and *ACO* comprise sizable multigene families in plants, and multiple internal and external stressors can influence various members [39,40]. Downstream reaction genes are expressed when ET binds to its receptor ETHYLENE RECEPTOR 1 (ETR1) [41]. CONSTITUTIVE TRIPLE RESPONSE 1 (CTR1) is an ER-localised Raf-like protein kinase activated by the ET receptor without ET [42,43]. Activated CTR1 phosphorylates another ER-localised protein, ETHYLENE INSENSITIVE 2 (EIN2), and this phosphorylation prevents further ET signalling [41,42,43]. However, CTR1 becomes inactivated in the presence of ET, and CTR1-mediated phosphorylation of EIN2 is reduced, resulting in the cleavage of the EIN2 c-terminal, which moves into the nucleus and activates the transcription factor ETHYLENE INSENSITIVE 3 (EIN3) and its homologue EIN3-LIKE 1 (EIL1) which are the primary mediators of the transcriptional responses to ET. EIN3/EIL1 also increases the expression of *ETHYLENE RESPONSE FACTORS (ERFs)* [44,45], causing plants to exhibit stress-related reactions mediated by ET [46]. It has been demonstrated that *ERFs* are responsible for regulating plant immunity [47,48], metabolic and morphological adaptations to flooding [49], the expression of mechanisms to scavenge reactive oxygen species, and the alteration of enzyme activity under salinity and heavy metal stress conditions [50,51].

It has been shown in numerous experiments that ET has additional functions in the regulation of plant defences against chewing insect herbivores and phytopathogens [52,53]. The chewing of herbivores induces ET production, which functions synergistically with JA to induce plant defences [54]. ET increases plant resistance to herbivory and can indirectly affect the insect’s physiology [55]. Several JA biosynthesis enzymes, such as lipoxygenase and allene oxide cyclase, are produced by ET-mediated defence induction in tomatoes [53]. Casteel et al. [56] and Bak et al. [57] showed that aphid-transmitted potyviruses suppress anti-insect defences via ET signalling. Similarly, another study on aphid infestation demonstrated that the R-gene-mediated aphid resistance in *Medicago truncatula* does not require ET signalling [58], despite the ethylene-insensitive mutant having moderate aphid resistance, indicating that ET negatively regulates the resistance. In an *ACS*-related study, the effect of ET on herbivory differed depending on the herbivore, whereby rice expressing antisense *ACS* showed a decrease in resistance to the Asiatic rice borer (*Chilo suppressalis*) but an increase in resistance towards the brown planthopper (*Nilaparvata lugens*) [59,60]. In summary, this indicates that ET regulates the herbivory resistance of plants either positively or negatively depending on the herbivores and plants.

## 4. Crosstalk between ET and Other Phytohormones in Insect Herbivory

**Figure 3:**
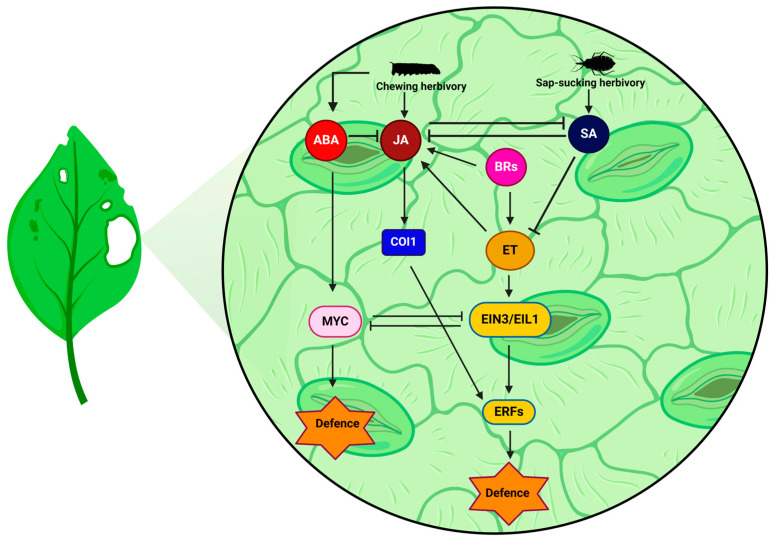
This scheme represents the signalling networks of the plant defence hormones ET, SA, JA, ABA, and BRs in a simplified manner. JA is activated upon the chewing insect herbivory, and COI1 allows further JA signalling to occur, resulting in the activation of *ERFs* with ET and suppression of the SA response. ERF induces the gene responsible for herbivory immunity. Chewing herbivory also induces ABA, which induces *MYC* and activates herbivory defence-related genes, while ABA represses JA at the same time. Additionally, *MYC* and *EIN3* are induced by ABA and ET, respectively, and antagonistically regulate each other. BRs induce the ET biosynthesis pathway and may positively regulate JA signalling, inducing an ERF-mediated herbivory response. Unlike chewing herbivores, sap-sucking herbivores do not induce JA but stimulate SA by minimising damage to plant tissues and their elicitors. SA suppresses both JA and ET, thus preventing plants from initiating an ERF-mediated herbivory response. Abbreviations: ET, ethylene; SA, salicylic acid; JA, jasmonic acid; ABA, abscisic acid; BRs, brassinosteroids; ERFs, Ethylene Response Factors; COI1, Coronatine Insensitive 1, MYC, MYC-related transcription activator.

Phytohormone-mediated signal transduction pathways activate plant defensive responses against insect herbivory [61]. There are studies that have shown the regulation of ET-mediated insect herbivory independent of other phytohormones. For example, solar UV-B radiation-induced ET increases isoflavonoid malonyl genistin and reduces *Anticarsia gemmatalis* larvae leaf consumption in soybean [62]. Similarly, a study on stink bug soybean herbivory found that treating ET precursor *ACC* induces isoflavonoids that inhibit a stink bug’s growth, but treating other hormones such as JA and SA did not increase the isoflavonoid contents [63]. Also, a study on maize plants and a phloem sap-sucking maize leaf aphid found that ET regulated the accumulation of transcripts of *MAIZE INSECT RESISTANCE 1 (MIR1)* in maize, independently of JA, which increased the level of resistance aphids [64]. However, in most cases, the plant response to stress, including insect herbivory, requires fine-tuned simultaneous regulation of multiple phytohormone networks. Several phytohormones, such as JA, SA (salicylic acid), ET, and abscisic acid (ABA), have been extensively investigated for their roles in protecting plants from pathogens and insect herbivory [65,66]. Through its participation in a complex signalling network with JA, SA, and ABA, ET is essential in activating plant defences against various biotic stressors (Figure 3) [67,68].

### 4.1. JA and ET Synergically Help Plants Resistant to Herbivory

In particular, ET and JA are known to act synergistically and agonistically [69,70]. The stress response is activated when plants are attacked by necrotrophic fungi or insects that generate JA/ET signalling [71]. These two hormones regulate the immune response and defence [72]. ERFs target JA/ET-responsive gene promoters for transcriptional activation or repression [47]. For example, *ETHYLENE RESPONSE FACTOR 1 (ERF1)*, a transcriptional element for the ET response, has expression positively regulated by JA in *Arabidopsis* [73,74]. The EIN3 and EIL1 TFs also stimulate *ERF1* and *OCTADECANOID-RESPONSIVE ARABIDOPSIS 59 (ORA59)* transcription, activating the JA pathway via the ERF branch, as ET stimulates EIN3/EIL1 protein accumulation [75]. This activation supports the defence against pathogens by activating the JA-responsive marker gene *PLANT DEFENSIN 1.2 (PDF1.2)*. Furthermore, Hu et al. [53] demonstrated that ERF15 and ERF16 can trigger the rapid release of JA when herbivores attack. Recent insights by Pieterse et al. [76] and Nishad et al. [77] showed that JA and ET interact to enhance defences against pathogens and herbivores. This indicates that JA and ET synergistically increase the pathogen resistance of plants. However, there are examples where the two hormones are antagonistic. Several molecular studies have shown that JA-activated ABA-responsive transcription factor MYC2 and ET-stabilised EIN3/EIL1 mutually reduce their activity. A weakening of *MYC2*’s repression to defend against generalist herbivores caused by EIN3/EIL1 interaction with MYC2 represses the wound-responsive gene *VEGETATIVE STORAGE PROTEIN 2 (VSP2)* and the herbivore-induced gene *CYP79B3* via JA signalling [72,78]. This shows that JA and ET may also regulate the plant defence antagonistically. Similarly, another study on plant development also showed an antagonistic relationship between JA and ET. In detail, the hooked hypocotyl found in ET-treated etiolated *Arabidopsis* seedlings is repressed by JA-induced EIN3 BINDING F-BOX PROTEIN 1 (EBF1), which facilitates EIN3/EIL1 degradation while JA-induced MYC2 inhibits EIN3 activity [74]. This indicates that ET may promote the synthesis of protective proteins, secondary metabolites, and other volatile organic molecules by regulating the expression of various genes involved in the plant defence against herbivory, most likely by synchronising JA signalling [79,80]. However, another study on the maize leaf aphid by Louis et al. [64] showed an increase in ET-mediated aphid resistance independent of JA. This shows that, in most cases, ET and JA together regulate resistance responses to herbivory in plants.

### 4.2. SA Suppresses JA/ET during Herbivory

Like JA, SA is another hormone that plays an essential role in plant defences. While JA is more associated with necrotrophic pathogens and chewing herbivores, the SA response participates in defence responses towards biotrophic pathogens and sap-sucking insects [81,82]. In general, SA is known to have an antagonistic relationship with JA/ET in plant immunity regulation. For example, the key regulator of the JA/ET response *ORA59* is transcriptionally suppressed, and ORA59 protein accumulation is reduced in the presence of SA, thus suppressing the JA/ET response [83,84,85]. Interestingly, a recent study identified that the mechanism behind SA-mediated ORA59 protein reduction is independent of *ORA59* transcription, which suggests that EIN3 interacts with ORA59 and degrades ORA59 [86]. This indicates that SA regulates the abundance of the ORA59 protein and affects SA and JA/ET crosstalk. However, SA does not always repress the JA/ET response; sometimes, it induces JA/ET. A recent study suggested that SA could promote a RESISTANCE TO P. SYRINGAE 2 (RPS2)-mediated JA response by degrading JA response repressor JASMONATE-ZIM-DOMAIN PROTEIN (JAZ) via NONEXPRESSOR OF PATHOGENESIS-RELATED GENE 3 (NPR3) and 4 (NPR4) [87]. While SA inhibits the JA/ET response by regulating the transcription factor of the response, ET also suppresses the SA response. Inhibition of SA in the JA/ET response was significantly reduced in *Arabidopsis* mutant *cev1* (*constitutive expression of VSP1*), which expressed the JA/ET response constitutively [88]. This indicates that plant sensitivity to SA is reduced when the JA and ET responses coincide.

Interestingly, some sap-sucking insects have an antagonistic relationship with SA and JA/ET. Several previous studies on herbivory of the sap-sucking insect whitefly (*Bemisia tabaci*) found that the fly’s larvae secrete an SA-activating elicitor, suppressing the JA-mediated defence mechanism [89,90]. Similarly, another previous study also suggested that, unlike chewing insects, sap-sucking insects such as aphids and whiteflies may be able to avoid chemicals produced by JA/ET, minimising the rupture of tissues [81]. However, in the case of thrips (Thysanoptera), which are cell-content-sucking insects, it also suppresses the JA response by inducing the SA response even though it physically damages plant cells [91]. Also, ET induces the SA response in this case, which is not commonly observed in typical insect herbivory [91]. This explains why SA generally suppresses the JA/ET response; thus, plants become susceptible to insect herbivory, and some sap-sucking insects take advantage of this crosstalk. However, as shown by Louis et al. [64], sap-sucking herbivory could induce the ET defence response independent of JA, and cellular rupture by herbivore thrips induces SA [91]; as such, there are unexplainable cases with the current knowledge.

### 4.3. Prospects and Potential of BRs in ET-Mediated Insect Herbivory

Brassinosteroids (BRs) respond to both abiotic and biotic stresses [92]. At this point, some studies have explored the BR-mediated herbivory response. One study using a tomato BR-deficient mutant (*dpy*) found an increase in trichome density in the mutant, indicating that BRs may negatively affect herbivory [93]. At the same time, the same study found that the mortality of the chewing herbivore *Spodoptera frugiperda* increased in the same mutant, while the JA-insensitive mutant (*jai1-1*) reduced the mortality, indicating that BRs negatively regulate the JA-mediated herbivory pathway [93]. In contrast, a study using tobacco (*Nicotiana attenuata*) with *BRASSINOSTEROID INSENSITIVE 1 (BRI1)* (a BR receptor) silenced found a decrease in some of the defensive secondary metabolites in the plant, caused by a decline in active JA [94].

These studies imply that BRs may directly or indirectly play a role in herbivory-related mechanisms. However, understanding the full scope of the mechanisms behind BR-mediated anti-herbivory remains challenging, as comprehensive studies are still needed. Although studies on BR-mediated herbivory are lacking, some pieces of the puzzle connect ET and BRs, which will clarify and improve our knowledge of unknown BR-regulated herbivory pathways. Some studies on the ET biosynthesis pathway confirmed that BRs play a positive role in regulating the *ACS,* which synthesises the ET precursors. In detail, BRs increase ethylene biosynthesis by stabilising the ACS protein and inducing *ACS* transcriptionally [95,96]. Although with the current knowledge, the mechanism behind BR-mediated ACS stabilisation is not fully understood, a recent study has illuminated some parts of BR-mediated ACS stabilisation. In detail, ACS5 forms a complex with E3 ligase SEVEN IN ABSENTIA OF ARABIDOPSIS 2 (SINAT2) and E3 ligase ETHYLENE OVEREXPRODUCER 1 (ETO1) during normal conditions, and two of the ligases destabilise ACS5 [97]. In the presence of BRs, 14-3-3 protein also binds to the ACS5/SINAT2/ETO1 complex, and the interaction degrades SINAT2 and ETO1, leaving ACS5 behind [97]. As a result, ACS5 abundance increases in the system-inducing ET. Interestingly, several researchers found that SINAT2 regulates autophagy, which is a self-eating pathway in organisms activated during nutritional and drought stress [98,99]. Although the effect of SINAT2 on herbivory has not yet been determined, this indicates that in the presence of BRs, autophagy may be positively regulated. Thus, plants could be more resistant to herbivory. Also, a recent study suggests that EIN3, a key factor in ET signalling, interacts with BRASSINOZALE RESISTANT 1 (BZR1), the key regulator of BRs’ response, and induces *HOOKLESS 1 (HLS1)* and *PACLOBUTRAZOL RESISTANCE FACTORs (PREs)*, causing apical hook development in the etiolated seedlings of *Arabidopsis* known as a critical phenotype of ET [100]. Likewise, studies linking BRs and ET were focused on either the biochemical aspect or abiotic stress aspect, with little emphasis on biotic stress. However, these ET and BR studies imply a strong chance that BRs may positively regulate ET-mediated herbivory simultaneously. Therefore, more studies are needed to experimentally confirm the mechanism behind the BR-mediated herbivory response.

Here, we have discussed several studies on insect herbivory involving ET and other hormones such as JA, SA, and BRs. These studies show that the connection between the ET-mediated defence and various hormones is complex and varies depending on the plant and insect involved. Further research is needed to uncover the pathways and mechanisms underlying the ET-mediated herbivory response.

## 5. Ethephon Applications in Herbivory Studies

Plants have a low ET content under normal conditions [101,102]. ET is linked to plant defence responses when treatments with external ET or its precursors are used [103]. Many studies have used ACC, ET gas, and ethephon (ETH) (2-chloroethane phosphonic acid) to induce the ET response. Among the three chemicals, ET-response-inducing ETH has been widely used in commercial agriculture because it is cost-effective and relatively more straightforward than ET gas [104]. In detail, ETH releases ET through its decomposition and thus can be used to replace ET in crop production [102,105]. For example, ETH is used as a herbicide to remove weeds such as Messina creeper (*Ipomoea cairica*) and promote pineapple (*Ananas comosus*) production by inducing flowers [106,107]. Also, many studies have found that ET-induced phenomena, such as fruit ripening, are dependent on the dosage of ETH, which is very similar to natural ET [108].

Because of the similarity between ET gas and ETH, many studies on stress, including herbivory studies, have used ETH as a substitute. For example, a study on rice using ETH showed improvements in carbohydrate metabolism, which could influence responses to biotic stresses like herbivory [109]. External use of ETH enhanced tomato fruitworm (*Helicoverpa zea*) growth in tomatoes and interfered with the wounding response by reducing *PROTEINASE INHIBITORS 2 (PIN2)* and *POLYPHENOL OXIDASE (PROF)* expression [110]. ETH has also been widely used to confirm the function of ET in response to pathogens. For instance, a study in soybean on the response to fungal attack by *Fusarium virguiforme* found that ET biosynthesis genes like *ACS* and *ACO* were induced when ETH was applied [111]. A study on a fungus (*Erysiphe necator*) that causes powdery mildew in grapes found a possible antagonistic effect, operating between ET and JA, on the resistance to ETH [112]. This indicates that ETH could be used as an alternative to ET in studies on herbivory and other forms of plant stress.

## 6. Conclusions and Prospects

The association between ET and plant stress resistance is recognised. Previous research has found that ET is essential in the plant’s response to biotic stresses. Furthermore, research indicates a link between ET signalling, the accumulation of defence-related genes, and insect herbivory resistance in response to ET treatments. Although some studies have addressed the role of ET in inducing resistance against biotic stress in plants, few have addressed insect herbivory. The next step will be to investigate the role of ET biosynthesis, signalling, and pathways in response to insect herbivory in crops. Elucidating ET’s role in insects and other herbivorous ecology in plant self-protection systems means that ET can be extensively utilised as an economical, eco-friendly, and functional chemical for pest management. Thus, it could potentially lead to a new era of pest management that is free from conventional pesticides and their adverse environmental and health effects. Using the ethylene-inducing chemical ETH could reduce the potential challenges of conducting such studies. Since ETH is economical and relatively more convenient to handle, it can be used as a trigger to release volatile signals that may help study inter-plant communication in response to herbivory. Additionally, ET could be employed in breeding programs involving the field performance of laboratory-selected resistance traits. We believe that a breeding program incorporating knowledge and studies of ET-mediated herbivory in diverse plant species could become a promising solution for the generation of durable field herbivory-resistant crops.

The study of ET-mediated herbivory stress responses in plants is promising to help us understand plant–insect interactions and develop sustainable pest management practices.

## Data Availability

This study did not create or analyse new data, and data sharing does not apply to this article.
